# Unraveling the Evolution of Dynamic Active Sites of LaNi_*x*_Fe_1–*x*_O_3_ Catalysts During OER

**DOI:** 10.1021/acsami.4c02502

**Published:** 2024-04-22

**Authors:** Haritha Cheraparambil, Miquel Vega-Paredes, Christina Scheu, Claudia Weidenthaler

**Affiliations:** † Max-Planck-Institut für Kohlenforschung, Kaiser-Wilhelm-Platz 1, Mülheim an der Ruhr 45470, Germany; ‡ Max-Planck-Institut für Eisenforschung, Max-Planck-Straße 1, Düsseldorf 40237, Germany

**Keywords:** perovskites, oxygen evolution reaction, active sites, surface-enhanced raman spectroscopy, electron energy loss spectroscopy

## Abstract

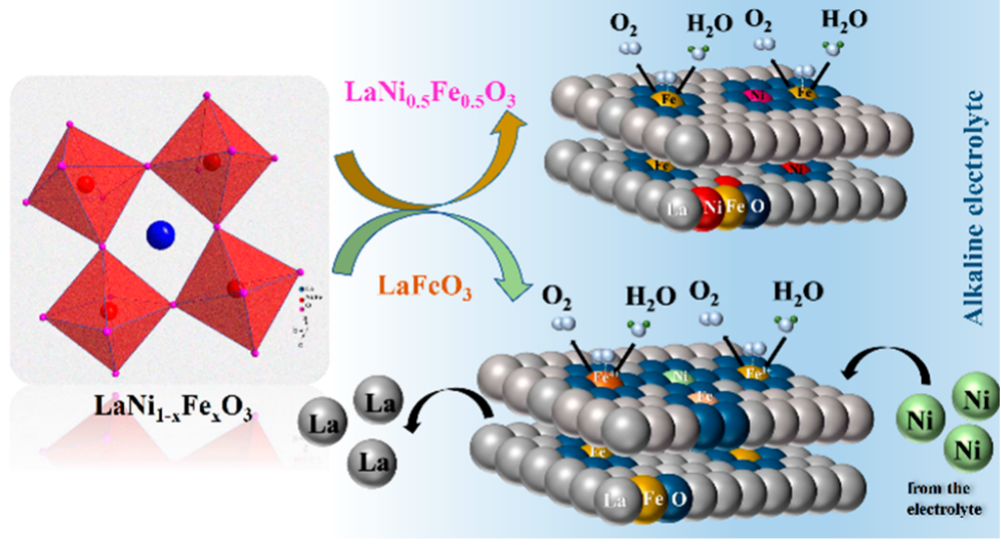

Perovskites have attracted tremendous attention as potential catalysts for the oxygen evolution reaction (OER). It is well-known that the introduction of Fe into rare earth perovskites such as LaNiO_3_ enhances the intrinsic OER activity. Despite numerous studies on structure–property relationships, the origin of the activity and the nature of the active species are still elusive and unclear. In this work, we study a series of LaNi_*x*_Fe_1–*x*_O_3_ perovskites using in situ electrochemical surface-enhanced Raman spectroscopy and electron energy loss spectroscopy to decipher the surface evolution and formation of active species during OER. While the origin of the activity arises from NiOOH species formed from the active Ni centers in LaNiO_3_, our work shows that Fe serves as the active center in LaNi_0.5_Fe_0.5_O_3_ and forms Fe–O–Ni and FeOOH species during OER. The OER activity of LaFeO_3_ originates from FeOOH species, which interact with the soluble Ni species in the electrolyte forming an active electrode–electrolyte interface with high-valent stable surface iron species (Fe^4+^) and thereby improving the performance. Our work provides deeper insights into the synergistic effects of Ni and Fe on the catalytic activity, which in turn provides new design principles for perovskite catalysts for the OER.

## Introduction

The growing demand for energy has attracted tremendous attention on hydrogen as a potential fuel for the future.^1,2^ One of the cleanest methods to produce hydrogen is water splitting.^3^ It involves two counter-reactions, hydrogen evolution on the cathodic and oxygen evolution on the anodic side. Oxygen evolution is a sluggish reaction, as it involves a four-electron transfer. Therefore, much effort has been devoted to the development of efficient and active OER catalysts.^4,5^ Among the affordable, accessible, and potential electrocatalysts, simple perovskites with the formula ABO_3_ have proven to be viable options. Owing to their chemical tunability and stability, they are often used for alkaline oxygen evolution reactions.^5−9^

LaNiO_3_ has shown promise as an electrocatalyst due to its high Ni–O covalency and occupancy of the *e*_g_ valence band (equal to 1).^10−12^ The physicochemical properties of perovskites have been tuned by several design strategies, including strain engineering,^10,13^ variation of stoichiometry^14,15^ and defect engineering^16,17^ to enhance the OER performance of the catalysts. Recent studies show that the substitution of Ni with Fe at the B site boosts the OER performance of LaNiO_3_.^18−22^ Wang et al. have synthesized a series of LaNi_*x*_Fe_1–*x*_O_3_ with coral morphology and found that LaNi_0.8_Fe_0.2_O_3_ has the best OER performance with a Tafel slope of 102.8 mV/dec,^22^ whereas the same perovskite composition yielded better OER activity with nanorod morphology as reported by Wang et al.^18^ The OER performance of the LaNi_*x*_Fe_1–*x*_O_3_ was improved by a rational design of the interfaces between the crystalline and the amorphous phases.^19^ Furthermore, Wang et al. performed ab initio modeling to infer the role of Fe in boosting the OER performance of LaNiO_3_. They reported that high-valent Fe cationic species form Ni–O–Fe bridges and enhance the TM 3d–O 2p hybridization, thereby increasing the OER activity.^20^ Despite numerous studies on the fundamental, structural, and electronic bulk properties of LaNi_*x*_Fe_1–*x*_O_3_, information on the origin of the activity, the nature of the active species, and the role of the A site is still elusive and unclear. Although there is much evidence for the influence of the electrolyte impurities on the active Ni sites,^23−25^ the dependence of the soluble Ni species in the electrolyte on the active Fe sites is challenging and still under discussion.^26−28^ Ye et al. have reported the effect of Ni and Fe on Ni(OH)_2_/NiOOH films. They found out that the presence of Ni impurities hindered the electrocatalytic performance.^28^ However, the response of the active Fe site in the presence of Ni impurities is still elusive. In addition, several studies on understanding the reaction mechanism of perovskites are limited to the surface reconstruction during OER.^29−31^ While the true nature of active sites in hydr(oxy)oxides is still under discussion,^32,33^ the structural complexity of perovskites makes it even more difficult to probe the short-lived dynamic species. For a deeper understanding of the role of the catalyst in the OER mechanism, a combination of in situ/operando and ex situ characterization studies of the reaction intermediates are indispensable. In this context, surface-enhanced Raman spectroscopy (SERS) has emerged as a powerful tool for studying the interfaces due to its specific fingerprints and high sensitivity.^34^

In this work, we investigate the synergistic effect of Fe and Ni in a series of LaNi_*x*_Fe_1–*x*_O_3_ perovskites and the influence of Ni electrolyte impurities on LaFeO_3_ using in situ electrochemical surface-enhanced Raman spectroscopy (SERS) and electron energy-loss spectroscopy (EELS) during the OER. We provide direct spectroscopic evidence for the surface evolution and the nature of short-lived active species of LaNi_*x*_Fe_1–*x*_O_3_ during the OER. In addition, the systems are studied at the local and bulk levels using total X-ray scattering and subsequent pair distribution function (PDF) analyses. This work leads to a deeper understanding of the structure–property relationships and the origin of catalytic activity in Ni–Fe-based perovskites, which will help in the development of catalysts for alkaline OER.

## Results and Discussion

### Bulk and Local Structure Analysis

**Figure 1:**
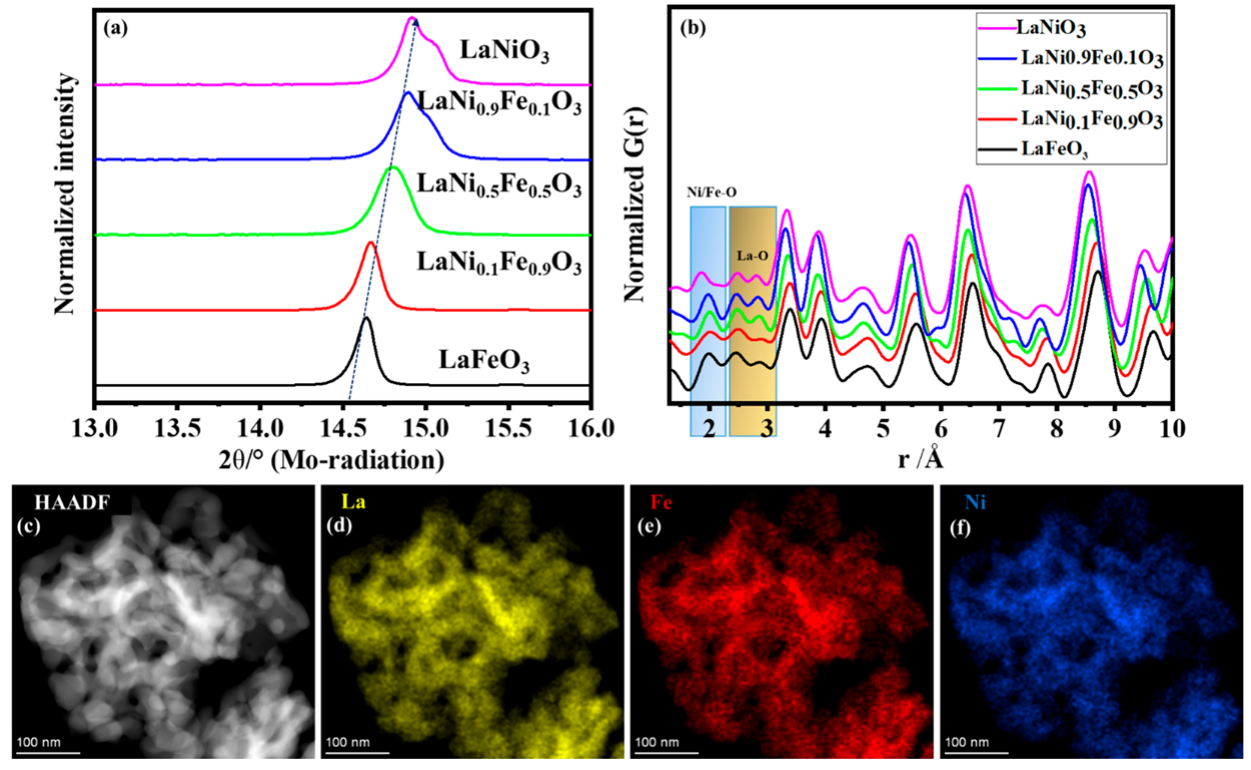
(a) Shift of the peak position between 14.5 and 15.5° 2θ for different LaNi_*x*_Fe_1–*x*_O_3_ perovskites, the arrow shows the shifts. (b) Short-range experimental PDFs of LaNi_*x*_Fe_1–*x*_O_3_ with Ni/Fe–O pair correlation marked by a blue square and La–O pair correlation marked by a yellow square. (c) HAADF image and (d), (e), and (f) composition maps of La, Ni, and Fe of LaNi_0.5_Fe_0.5_O_3_.

A series of simple perovskites were synthesized via a modified solution combustion method followed by calcination.^35^ The perovskites were tuned by changing the composition of the B site (LaNiO_3_, LaNi_0.9_Fe_0.1_O_3_, LaNi_0.5_Fe_0.5_O_3_, LaNi_0.1_Fe_0.9_O_3_, and LaFeO_3_). The samples are studied by X-ray diffraction and total scattering experiments. The X-ray diffraction patterns are shown in Figure S1a. The main reflection around 14–15.5° 2θ is shifted to lower angles with increasing Fe content, as shown in Figure 1a. Thus, the unit cell volume increases with the Fe content, which is attributed to the larger ionic radius of Fe compared to that of Ni.^36^ The fwhm of the reflections decreases from LaNiO_3_ to LaFeO_3_ (as seen for the main reflection around 14–15.5° 2θ in Figure 1a), which indicates larger crystallite sizes with increasing Fe substitution in the perovskites.

Experimental PDFs of LaNi_*x*_Fe_1–*x*_O_3_ are compared in the short-range (Figure 1b) as well as in the long-range (Figure S1b). The maximum distance at which peaks are observable gives information about domain sizes.^37^ LaNiO_3_ has a domain size of ∼50 Å and this increases to ∼100 Å in LaFeO_3_ as shown in Figure S1b. As Figure 1b depicts, the first pair correlation marked with a blue square corresponds to the distances between Ni/Fe–O in perovskites. For a perfect LaNiO_3_ crystal structure, the Ni–O pair correlation appears at 1.94 Å. However, the as-synthesized LaNiO_3_ has a shorter Ni–O bond length of 1.86 Å, and Fe substitution increases the bond length (Ni/Fe–O) to 1.98 Å in LaNi_0.5_Fe_0.5_O_3_, and further to 2 Å (Fe–O) in LaFeO_3._

A similar observation is found in the next pair correlation marked with a yellow square, which gives information about La–O distances in a 9-fold coordinated polyhedron. These observations are further attributed to the larger ionic radius of Fe^3+^ compared to that of Ni^3+^. These experimental results support the theoretical DFT calculations reported by Wang et al.^20^ explaining that the increased bond length leads to increased Coulomb repulsion inducing a bandgap opening between occupied Ni 3d/O 2p and unoccupied Fe 3d/O 2p hybridized bands, reducing the TM 3d bandwidth and weakening the TM 3d/O 2p hybridization.

The Rietveld refinements and the PDF refinements obtained for all samples are shown in Figure S2 and Figure S3, respectively. The structures with Fe substitution ≤50 wt % (LaNiO_3_, LaNi_0.9_Fe_0.1_O_3_, LaNi_0.5_Fe_0.5_O_3_) crystallize in rhombohedral symmetry (space group *R*3*c*), whereas with increasing Fe substitution >50 wt % (LaNi_0.1_Fe_0.9_O_3_, LaFeO_3_) the structures change to orthorhombic symmetry (space group *Pbnm*). All compositions are phase pure except for LaNiO_3_ and LaNi_0.9_Fe_0.1_O_3_ which have trace amounts of NiO, <1 and ∼2 wt %, respectively. The symmetry change is also observed with a shoulder (between 14.7 and 15.0° 2θ) developing with the increasing Ni content, as shown in Figure 1a. The refined parameters are summarized in Table S1 and Table S2.

High-resolution transmission electron microscopy of LaNi_0.5_Fe_0.5_O_3_ shows that the particles are crystalline, densely packed, bulky in nature, and have a platelet-like morphology (Figure S4). The EDS maps also confirm the uniform distribution of the elements, as shown in Figure 1c–f. The particle size increases from LaNiO_3_ to LaFeO_3_, which is in good agreement with the observations from XRD and PDF.

All samples were characterized by Raman spectroscopy (using a 523 nm laser). The Raman spectra of LaFeO_3_ and LaNi_0.1_Fe_0.9_O_3_ are in good agreement with the previous reports of structures with orthorhombic symmetry (space group of *Pbnm*).^38,39^ The Raman modes of LaNiO_3_, LaNi_0.9_Fe_0.1_O_3_, and LaNi_0.5_Fe_0.5_O_3_ are similar to the previously reported structures crystallizing in rhombohedral symmetry (space group *R*3*c*).^40−42^ All of the Raman spectra are shown in Figure S5.

The as-prepared LaNiO_3_ has four distinct modes, one at 156 cm^–1^ corresponding to the E_g_ symmetry arising from the internal La vibrations in the hexagonal plane, one at 200 cm^–1^ corresponding to the rhombohedral distortion with respect to the tilt angle of the octheadra with A_1g_ symmetry, and the two modes around 400 and 450 cm^–1^ corresponding to the bending and anti stretching vibrations of the NiO_6_ octahedra with E_1g_ symmetry. With 10% substitution of Fe in LaNiO_3_, the bands below 200 cm^–1^ corresponding to La vibrations are less intense, and the high-frequency mode around 400 cm^–1^ is quite broad. This indicates that the substitution of Ni by Fe induces local disorder in the vibrations of the NiO_6_ octahedra. There is a new broad feature at around 570 cm^–1^, which is resonantly enhanced with the increased Fe substitution (LaNi_0.9_Fe_0.1_O_3_, LaNi_0.5_Fe_0.5_O_3_, and LaNi_0.1_Fe_0.9_O_3_). A complex oxide like LaFeO_3_ has 24 Raman active modes; therefore, it is difficult to observe all of them experimentally. In our LaFeO_3_ system, the modes observed below 200 cm^–1^ correspond to the La-site vibrations. The modes around 260 cm^–1^ are associated with the tilt vibrations of FeO_6_ octahedra, and the mode around 413 cm^–1^ corresponds to the bending vibrations of FeO_6_ octahedra with A_g_ symmetry. The modes between 470 and 500 cm^–1^ correspond to asymmetric stretching due to Jahn–Teller distortions with A_g_ symmetry. The broad mode centered at around 630 cm^–1^ is assigned to the B_1g_ mode, which corresponds to the symmetric stretching vibrations of the FeO_6_ octahedra. The additional modes beyond 700 cm^–1^ are assigned to multiphonon processes. LaNi_0.5_Fe_0.5_O_3_ has vibrational modes centered around 150, 228, 306, 400, 475, and 570 cm^–1^. The mode around 150 cm^–1^ originates from A-site vibrations, and the rest of the bands could be assigned to the vibrational modes corresponding to Fe–O and Ni–O octahedra. Similarly, LaNi_0.1_Fe_0.9_O_3_ has modes centered around 120, 176, 300, 386, 471, and 577 cm^–1^, a shoulder centered around 630 cm^–1^, and a vibrational mode centered around 1320 cm^–1^ corresponding to multiphoton processes. The vibrational modes of the perovskites discussed above are briefly generalized in Table S3.

### Electrochemical Performance of LaNi_*x*_Fe_1–*x*_O_3_

**Figure 2:**
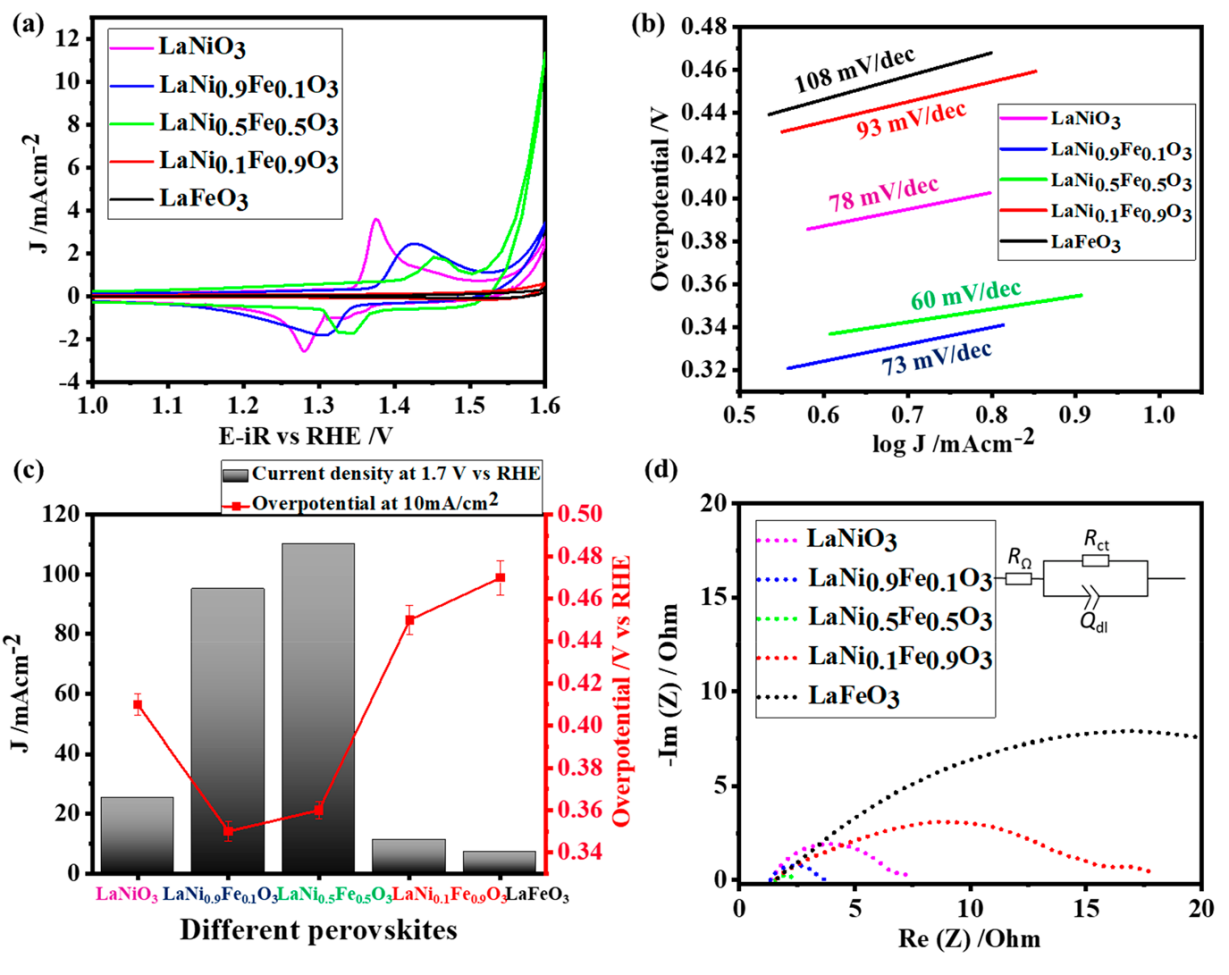
(a) Cyclic voltammetry, (b) Tafel slopes, (c) comparison of overpotentials at 10 mA/cm^2^, and current density at 1.7 V vs RHE, and (d) results of impedance spectroscopy measurements obtained for the different LaNi_*x*_Fe_1–*x*_O_3_ perovskite compositions.

All different compositions were tested for the alkaline oxygen evolution reaction following the well-defined protocols of Jaramillo and co-workers.^43^ The electrolyte used was commercial 1 M KOH unless otherwise specified. Cyclic voltammetry experiments were performed to study the redox behavior of the surfaces of the systems and show a clear trend of the effect of Fe substitution (Figure 2a). The activity increases until the Fe substitution reaches 50%, beyond, which the activity decreases. With the addition of Fe into the LaNiO_3_ structures, there is a shift toward a higher anodic potential in the precatalytic redox behavior, indicating the synergistic effect between Ni and Fe, consistent with previous reports on Ni–Fe oxides.^13,20,44^ After stabilization of the surface of the samples through 50 cycles, linear sweep voltammetry was performed, as shown in Figure S6a. LaNi_0.5_Fe_0.5_O_3_ outperforms the series of electrocatalysts and LaFeO_3_ has the lowest activity. A comparison of the overpotential at 10 mA/cm^2^ and current density at 1.7 V vs RHE was performed for different systems as shown in Figure 2c. The substitution of 10% Ni by Fe improves the current density by a factor of 4 and reduces the overpotential from 410 to 350 mV. A comparison of the Tafel slopes (Figure 2b) also confirms the superior activity of LaNi_0.5_Fe_0.5_O_3_ which has a Tafel slope of 60 mV/dec compared to the unsubstituted LaNiO_3_ with a Tafel slope of 78 mV/dec and LaFeO_3_ with 108 mV/dec However, more than 50% substitution of Ni by Fe has a detrimental effect on the catalytic activity. It suffers from low current density and a high overpotential, which could be attributed to the leaching effect of Fe.^45^ The ICP-OES data of the electrolyte after the OER show the presence of Fe (0.03 ppm) for LaFeO_3_. Impedance spectroscopy was performed to understand the charge transfer kinetics. A comparison of the Nyquist plots is shown in Figure 2d. The simplified Randles model (as shown in the inset of Figure 2d) was used to fit the data, and the results are summarized in Table S4. LaFeO_3_ has a higher charge transfer resistance, indicating slow electron transfer, while LaNi_0.5_Fe_0.5_O_3_ has the lowest charge transfer resistance.

The electrochemical measurements show that among all the perovskite compositions studied here, LaNi_0.5_Fe_0.5_O_3_ has the best OER performance. The stability of the catalyst was tested for 10 h and found to be stable, as shown in Figure S6b. Post-mortem HR TEM analysis confirms that the crystallinity is retained, and the morphology is also preserved (shown in Figure S7).

### Elucidation of the Nature of the Active Sites

The origin of the activity and the nature of the active species were studied via in situ electrochemically surface-enhanced Raman spectroscopy (SERS). The catalysts were drop cast on the electrochemically roughened Au substrates and examined at different potentials (1.0 to 1.5 V vs RHE) using a red laser. A high-purity semiconductor-grade electrolyte was used to avoid iron and Ni impurities. The Raman spectra collected at 1.5 V are compared in Figure S8. For LaNiO_3_, Ni is known to be the active site for OER and forms NiOOH species.^23,25^ At 1.5 V, the in situ Raman spectra show the presence of two bands at 480 and 560 cm^–1^, corresponding to the symmetric and antisymmetric vibrational modes of Ni–O in NiOOH with E_g_ and A_1g_ symmetry, respectively.^32^ In LaNi_0.9_Fe_0.1_O_3,_ the in situ Raman spectra collected at 1.5 V indicate the formation of NiOOH at the relatively lower frequencies of 474 and 554 cm^–1^ along with the formation of a small band around 460 cm^–1^. The band at 450 cm^–1^ is usually attributed to Ni(OH)_2_, but the presence of Fe can induce a shift in the wavenumber. The recent reports indicate that the band at 460 cm^–1^ exhibits A_g_ symmetry and can be assigned to the Fe/Ni–O–Ni environment.^46,47^ However, the direct observation of NiOOH indicates that the active species could be attributed mainly to Ni sites forming NiOOH.

**Figure 3:**
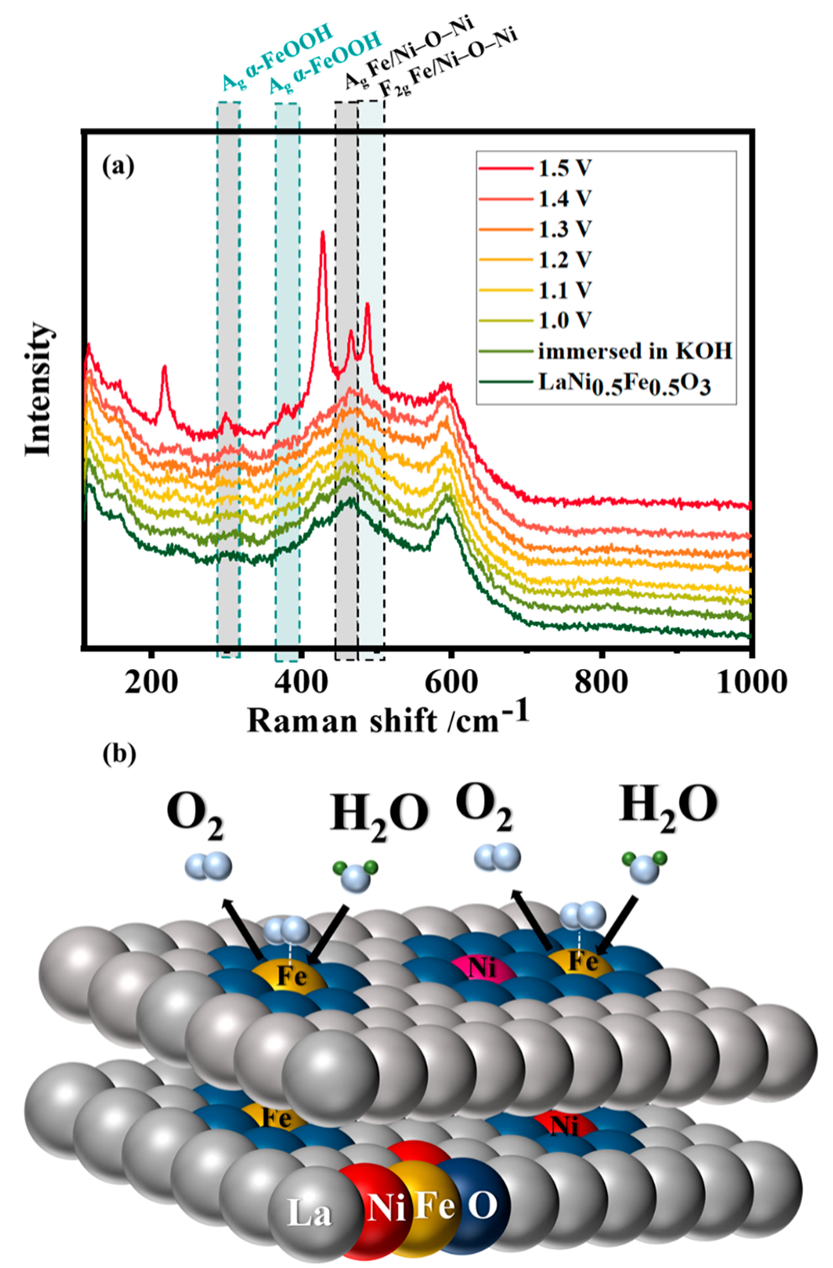
(a) In situ electrochemical SERS of LaNi_0.5_Fe_0.5_O_3_ in the potential range of 1.0 V to 1.5 V vs RHE in 0.1 M pure KOH (using 785 nm laser). (b) Pictorial representation of the surface evolution of LaNi_0.5_Fe_0.5_O_3_ during the OER to form the active species.

For LaNi_0.5_Fe_0.5_O_3_, where Fe and Ni occupy the B site equally, the in situ Raman data are shown in Figure 3a, and the Raman spectra measured at 1.5 V are compared in Figure S8. The perovskite is quite stable up to 1.4 V, as only negligible changes are observed. At 1.5 V, a dynamic surface reconstruction takes place. The new bands appear around 215, 300, 380, 424, 464, 486, and 580 cm^–1^. The bands around 300, 380, and 486 cm^–1^ correspond to the vibrational modes of α-FeOOH with A_g_ symmetry.^48,49^ The band around 480 cm^–1^ is attributed to the F_2g_ vibrations corresponding to the asymmetric bending of metal–oxygen bonds.^50^ The vibrational modes centered around 460 and 480 cm^–1^ indicate the presence of a Fe/Ni–O–Ni environment.^44,47^ The most prominent bands around 215 and 430 cm^–1^ correspond to the vibrations from La hydroxides and oxyhydroxides.^51^ These observations confirm that of the two possible active sites for the OER, Ni and Fe, Fe is the more prominent active site compared to Ni. This also does not exclude the possibility of a mixed Fe–O–Ni environment. However, no modes corresponding to NiOOH were observed, which is contrary to previous reports of NiFe oxides for OER.^32^Figure S9a and b shows the high-angle annular dark field (HAADF)-STEM images of LaNi_0.5_Fe_0.5_O_3_ before and after OER. The areas analyzed by EELS are highlighted by colored squares. However, only minor changes are observed in the EELS spectra collected before and after the OER (Figure S9c). This agrees well with the Raman spectra collected after the OER, which retain all the vibrational modes of the untreated perovskite (Figure S10). The surface evolution and active site formation are illustrated in Figure 3b. LaNi_0.5_Fe_0.5_O_3_ undergoes a dynamic surface evolution in which Fe is the most active site for the OER. Ni exists in a partially reduced state in a coordinated environment with Fe as Fe–O–Ni species. However, after OER the surface returns to its original state.

With further Fe substitution, in LaNi_0.1_Fe_0.9_O_3_, the vibrational modes formed at a bias of 1.5 V are shown in Figure S8. The bands centered around 300, 380, and 470 cm^–1^ are broad compared to those of LaNi_0.5_Fe_0.5_O_3_ and are typical for α-FeOOH. There is a new vibrational mode centered around 550 cm^–1^, which is also a characteristic feature for the surface evolution of LaFeO_3_ (described below) and corresponds to the formation of FeOOH.

**Figure 4:**
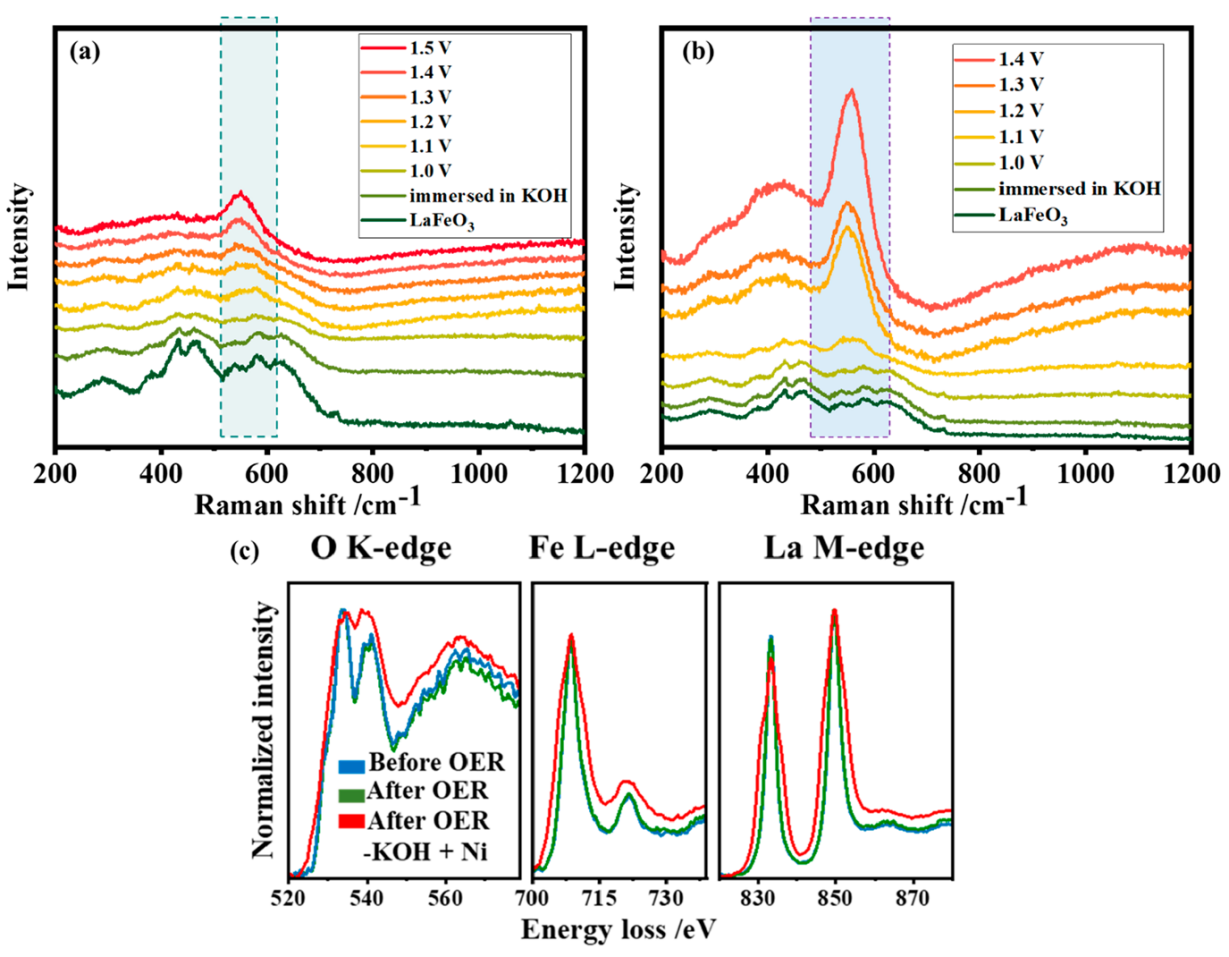
In situ electrochemical SERS (using a 785 nm laser) of LaFeO_3_ in the potential range of 1.0 V to 1.5 V vs RHE in (a) 0.1 M pure KOH and (b) 0.1 M pure KOH containing Ni as impurity. (c) Comparison of EELS spectra (O K-edge, Fe L-edge, and La M-edge) collected for LaFeO_3_ in different conditions: before OER (blue color), after OER in 1 M pure KOH (green color), and in 1 M pure KOH with Ni impurities (red color).

LaFeO_3_ perovskite was also studied by in situ SERS under different potentials (Figure 4a). The line width of the modes increases as soon as the sample comes into contact with KOH and becomes broader when a bias voltage is applied. The increased width indicates the local disorders. At about 1.2 V, a new intense and broad band around 545 cm^–1^ is observed, which becomes very prominent at higher potentials. Along with this, very broad and less intense bands appear between 200 and 500 cm^–1^. This indicates the formation of α-FeOOH.^52,53^ Unlike LaNi_0.5_Fe_0.5_O_3,_ the modes corresponding to La are not clearly distinguishable.

It is known that electrolyte impurities influence the activity of the catalysts.^28^ It is has been discussed that soluble Ni impurities are detrimental to the OER kinetics.^28,54^ We studied the effect of Ni impurities on LaFeO_3_. Figure S11 shows the cyclic voltammetry of LaFeO_3_ in 1 M KOH with different Ni impurities. The redox peaks of the perovskites become more pronounced as the amount of Ni impurity increases in the electrolyte, implying that the surface is more activated. Table S5 summarizes the different Ni contents in the electrolytes used, confirmed via ICP OES. This table also lists the concentration of Fe used in the electrolytes to rule out the influence of Fe on the activity of the catalysts. A comparison of the STEM images of the catalyst before and after the OER is depicted in Figure S12. It shows that with Ni impurities, the LaFeO_3_ particles become compacted during the OER and the porosity between the particles decreases. The presence of Ni impurities results in more aggregated particles and the formation of voids. On the one hand, the formation of cavities allows more KOH to penetrate and more active sites are exposed. On the other hand, this could also indicate a loss of material. However, strong aggregation could result in blockage of accessible active sites over longer cycles. The dynamics of Ni incorporation into LaFeO_3_ was studied by using in situ electrochemical Raman spectroscopy as shown in Figure 4b. As soon as a bias voltage is applied, the bands become broader, and at 1.1 V a band develops around 550 cm^–1^. This band intensifies with increasing potential, indicating that more active sites are generated. At 1.4 V, there are three separate broad bands around 299 cm^–1^, 400 cm^–1,^ and 550 cm^–1^, which correspond to the A_g_ vibrations of α-FeOOH.^49,55^ The reduction in Ni content in the electrolyte before (4.98 ppm) and after (1.91 ppm) the OER, as observed via ICP OES, confirms that some amount of the Ni impurity from the electrolyte enters the perovskite structure. However, the strong bands of NiOOH (∼470 and ∼550 cm^–1^) are not distinguishable, but the broader fwhm of the bands could also indicate the presence of other active species. The early formation and increase in the number of active species indicate that the presence of Ni impurities increases the OER activity of LaFeO_3._

The in situ Raman studies of LaFeO_3_ were complemented by EELS studies. Changes in the O K-edge, Fe K-edge, and La-M edge regions during the OER were tracked (Figure 4c, Figure S13). No changes were observed during OER in KOH. However, after OER in the presence of Ni impurities, significant changes appear in the EELS spectra. Three main features can be observed for the O K-edge: a prepeak around 530 eV which is attributed to the excitations from O 1s to 2p bands, a second peak around 535 eV, which corresponds to the hybridization of O 2p with La 5d, and a third peak around 540 eV to which mainly Fe 4sp type bands contribute. The O K-edge has two predominant peaks at ∼534 and 540 eV with different intensities initially and after catalysis in pure KOH, with the relative intensity of the second peak after OER being enhanced in the presence of Ni impurities. This change in relative intensity is due to a change in the electronic environment of the oxygen atoms of the LaFeO_3_ lattice, probably induced by a change in the oxidation state of the neighboring atoms. There is a strong suppression of the prepeak at ∼530 eV after OER in the presence of Ni impurities, which indicates the presence of oxygen vacancies.^56,57^

**Figure 5:**
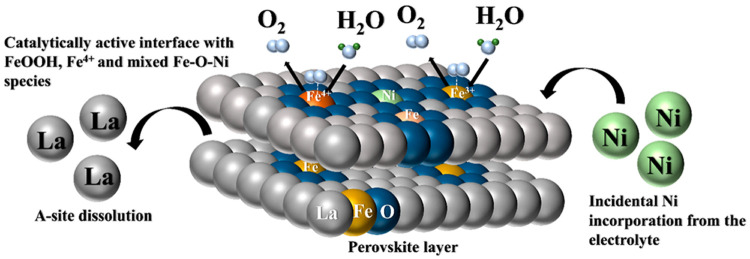
Pictorial representation of the surface evolution of LaFeO_3_ during the OER to form the active species.

In the pristine LaFeO_3_, the Fe L-edge consists of two peaks, L_3_ (708.5 eV) and L_2_ (721.5 eV) corresponding to the electron excitations from the 2p_3/2_ and 2p_1/2_ core states to unoccupied 3 d orbitals. The two peaks are separated by Δ*E* L_2,3_ = 13.2 eV due to the spin–orbit splitting of the Fe 2p_3/2_ and 2p_1/2_ states. In general, the energy difference between L_3_ and L_2_ peaks depends on the oxidation state, which is nominally 3+ in the pristine sample.^58−60^ Both the Fe L-edge and La M-edge regions undergo a significant broadening after the OER in the presence of Ni impurities. The thickness maps and zero-loss peaks of the three samples were checked to eliminate possible artifacts in the interpretation (Figure S14, S15). Each edge shows two shoulders, one on each side of the central peak. However, it is quite challenging to resolve the La M-edge and Ni L-edge because of their overlap. Nevertheless, the width indicates the presence of different environments and mixed oxidation states. The larger fwhm of the Fe-L_3_ peak can be attributed to a combination of Fe^3+^ species (centered around 708.5 eV), more oxidized species (∼710.6 eV), and more reduced oxidation states (∼707 eV). The presence of more oxidized Fe^4+^ species could indicate the presence of Fe^4+^=O bonds, as reported previously^61,62^ in systems with Fe as the active site. It could also indicate the presence of La vacancies as observed in previous reports.^63^ The leaching of La (0.09 ppm) was also confirmed by ICP-OES. The presence of a more reduced oxidation state could be attributed to the formation of oxygen vacancies^64^ and a mixed Fe–O–Ni environment^65^ during electrocatalysis. This in turn confirms the enhanced activation of LaFeO_3_ in the presence of Ni impurities. This is summarized in Figure 5.

The results show that in a series of LaNi_*x*_Fe_1–*x*_O_3_, LaNiO_3_ proceeds the OER activity through the formation of NiOOH species. When 10% of the B sites in LaNiO_3_ are substituted by Fe, as in the case of LaNi_0.9_Fe_0.1_O_3,_ Ni still acts as the main active center. However, as the Fe substitution increases to 50% and above, Fe becomes the active site. The surface reconstruction of LaNi_0.5_Fe_0.5_O_3_ is very dynamic, and the surface evolves into a combination of FeOOH and Fe–O–Ni species. However, these changes can be only be detected by in situ experiments. The OER activity of LaFeO_3_ is influenced by the Ni impurity in the electrolyte. The Ni impurity interacts with active Fe sites, resulting in morphological changes, dissolution of La, and the formation of oxygen vacancies. The early formation of a relatively large amount of active sites and the presence of oxidized Fe^4+^ species confirm the observation that Ni impurities enhance the OER activity of LaFeO_3._

## Conclusion

In summary, using a combination of in situ electrochemical SERS, EELS, and ICP OES measurements, we have elucidated the nature of the active sites of LaNi_*x*_Fe_1–*x*_O_3_ formed during OER. While LaNiO_3_ forms NiOOH as the active species during OER, we have demonstrated from the in situ spectroscopy data, that Fe is the active center in a mixed LaNi_0.5_Fe_0.5_O_3_ perovskite during OER. The activity of LaFeO_3_ originates from the active Fe center, which interacts with the Ni impurity in the electrolyte to forms more oxidized Fe^4+^=O species, which in turn increases the OER activity. However, LaFeO_3_ suffers from Fe leaching, which could hinder the activity over a larger number of cycles. Therefore, the interplay between Fe dissolution and the formation of stable active sites is crucial for a stable electrocatalyst. This interaction appears to be well balanced for LaNi_0.5_Fe_0.5_O_3_ and leads to the improved OER performance of this perovskite composition compared to the nonsubstituted perovskites, LaNiO_3_ and LaFeO_3_.

## Experimental Section

### Synthesis

La(NiFe)O_3_ perovskites were synthesized via a modified solution combustion method followed by calcination.^35^ To briefly explain the procedure, stoichiometric amounts of nitrates [La(NO_3_)_3_·6H_2_O from Alfa Aesar, CAS 10277–43–7, purity 99.99%; Ni(NO_3_)_2_·6H_2_O from Alfa Aesar, CAS 13478–00–7, purity ≥97%; Fe(NO_3_)_2_·9H_2_O from Sigma-Aldrich, CAS 7782–61–8, purity ≥98%] were dissolved in a mixture of water at RT. An equimolar molar amount of glycerol (Sigma-Aldrich, CAS 56–81–5) was added as the fuel, and the mixture was stirred at RT. After 15 min of stirring, 3 mL of nitric acid was added dropwise. The whole mixture was further heated to 250 °C until the solvent was completely evaporated and was followed by an autocombustion reaction. This resulted in the formation of spongelike precursor, and the so-obtained precursors were calcined at 600 °C for 3 h to obtain phase pure perovskites for LaNi_0.5_Fe_0.5_O_3,_ LaNi_0.1_Fe_0.9_O_3,_ and LaFeO_3_. LaNiO_3_ and LaNi_0.1_Fe_0.9_O_3_ were obtained after calcination of the precursors at 700 °C for 30 min, and they contain traces of NiO.

### Characterization

#### X-ray Powder Diffraction (XRPD)

Room temperature diffraction data were collected on a STOE STADI P transmission diffractometer equipped with a Mo radiation source (λ= 0.7093 Å) and a primary Ge (111) monochromator. Data were collected in the range between 15° and 40° 2θ with a scanning speed of 0.09° min^–1^ (step size, 0.015°) with a position-sensitive Mythen1K detector. Each sample was placed inside a borosilicate glass capillary (outer diameter 0.5 mm). All XRPD patterns were analyzed with the DiffracSuite Topas V6 software (Bruker AXS GmbH, Karlsruhe, Germany).^66^ The structure refinements were based on the crystal structure data taken from the Inorganic Crystal Structure Data Base (ICSD) LaFeO_3_ (ICSD 7794), LaNi_0.9_Fe_0.1_O_3_ (ICSD 84934), LaNi_0.5_Fe_0.5_O_3_ (ICSD 246033), LaNi_0.1_Fe_0.9_O_3_ (ICSD 84940), LaNiO_3_ (ICSD 93919), and NiO (ICSD 9866)_._

#### X-ray Total Scattering Data

Total scattering data were collected at room temperature in-house using a STOE STADI P diffractometer in transmission mode using Mo Kα radiation (λ = 0.7093 Å) equipped with a primary Ge(111) monochromator. The data were collected with a position-sensitive Mythen1K detector in the range between 5 and 120° 2θ with a step width of 0.015° 2θ. Data were further evaluated by pair distribution function (PDF) analysis using the program PDFgetX3^67^ within the package xPDFsuite.^68^*Q*_damp_ (0.027 Å^–1^) and *Q*_broad_ (0.003 Å^–1^) were determined with a silicon standard (Si NIST 640b). The structure refinement was based on the crystal structure data of LaFeO_3_ (ICSD 7794), LaNi_0.9_Fe_0.1_O_3_ (ICSD 84934), LaNi_0.5_Fe_0.5_O_3_ (ICSD 246033), LaNi_0.1_Fe_0.9_O_3_ (ICSD 84940), LaNiO_3_ (ICSD 93919), and NiO (ICSD 9866)_._

#### Electron Microscopy

Scanning transmission electron micrographs were acquired in a probe-corrected Titan Themis microscope (Thermo Fisher Scientific) operated at 300 kV. A 100 mm camera length was used, which resulted in a collection angle of 78–200 mrad for the high-angle annular dark field (HAADF) and 18–73 mrad for the annular dark field (ADF). Energy dispersive X-ray spectroscopy (EDS) and electron energy loss spectroscopy (EELS) images were acquired in the same microscope. For the EELS experiments, a dispersion of 0.250 eV per channel and a pixel acquisition time of 1 s were used. Transmission electron microscopy (TEM) images were collected with a H-7100 electron microscope from Hitachi operated at 100 kV acceleration voltage of LaB6 electron source, and the high-resolution transmission electron microscopy (HR-TEM) images were recorded with a Hitachi HF-2000 instrument equipped with a cold field emission gun (cold-FEG) operated at 200 kV. Scanning electron microscopy (SEM) images were taken on an ultrahigh-resolution cold field emission SEM Hitachi S-5500 operated at 30 kV.

#### Electrochemical Measurements

All electrochemical measurements were performed in a three electrode Teflon cell with a rotating disc electrode (Model: AFMSRCE, PINE Research Instrumentation), a reversible hydrogen electrode (HydroFlex, Gaskatel) as the reference, and a Pt wire as the counter electrode. 1 M KOH was used as the electrolyte unless specified. The temperature of the cell was maintained at 25 °C by a water circulation system. Prior to the measurements, the electrolyte was purged with argon to remove the dissolved oxygen from it. For the preparation of working electrodes, first of all, GC (PINE, 5 mm diameter, 0.196 cm^2^ area) electrodes were polished with an alumina suspension (5 and 0.25 μm, Allied High Tech Products, Inc.) and then washed in deionized water (DI) by sonication for 5 min. The catalyst ink was prepared by dispersing 4.8 mg of sample powder in 1 mL of mixed solution of DI water: Isopropanol (1:1) and 50 uL Nafion 117 (Sigma-Aldrich) binder and further sonicating for 30 min to form a homogeneous ink. 5.25 uL of catalyst ink (catalyst loading of 0.12 mg/cm^2^) was drop cast onto the polished glassy carbon electrode and dried under Argon atmosphere overnight. Cyclic voltammetry (CV) was performed at a scan rate of 50 mV/s within the 0.7 to 1.6 V vs RHE potential window. Linear scan voltammetry (LSV) was measured after stabilizing the surface via CV in a potential window of 0.7 to 1.7 V vs RHE at a scan rate of 10 mV/s. The data for the Tafel graphs were measured at the same scanning speed. The Tafel slope was derived from the equation η = *b* log *j* + *a*, where η, *b*, and *j* are the overpotential, Tafel slope, and current density, respectively. Chronopotentiometry was performed at 10 mA/cm^2^ of geometric current density in 1 M KOH. Electrochemical impedance spectroscopy (EIS) was measured at 1.66 V vs RHE and 5 mV of amplitude within the 100 mHz–100 kHz frequency range, and the obtained Nyquist plots were then fitted to the equivalent circuit model using the EC-Lab software. The IR drop was compensated at 85%.

#### In Situ Surface-Enhanced Raman Spectroscopy (SERS)

The spectra of powder samples were collected with an Invia Renishaw Raman microscope equipped with a laser excitation of 532 nm and 1800 l/mm grading and coupled with a 50× objective lens (Leica). In situ Raman study was done with 785 nm laser, 1200 l/mm grading, 50× objective lens (Leica) in 0.1 M high-purity semiconductor-grade KOH in homemade electrochemical cell. Au substrate was polished and roughened following the previously reported protocol^69^ and the sample ink solution was dropcasted on to it. The sample ink solution consisted of 4.8 mg of catalyst, 1 mL of water: isopropanol (3:1) mixture, and 50 uL of nafion. Pt wire and RHE were used as the counter and reference electrodes, respectively. Argon saturated electrolyte was used, and the flux was controlled using a peristaltic pump with a flow rate of 1–8 mL/min in order to remove the bubble formation. Before applying bias, the sample was immersed in electrolyte for 30 min to check the solvation effect. In situ Raman spectra were then collected with fixed potential in the chronoamperometric (CA) mode from 1.0 to 1.5 V vs RHE.

#### Inductively Coupled Plasma-Optical Emission Spectrometry (ICP-OES)

The measurements were carried out with a SPECTROGREEN instrument, and the electrolyte solution samples were taken from the electrochemical cell before and after the reaction.

## Supporting Information Available

Figure S1: (a) X-ray diffraction patterns and (b) long-range experimental PDFs of different LaNi_*x*_Fe_1–*x*_O_3_ perovskites; Table S1: Refined parameters obtained after the final Rietveld refinement of LaNi_*x*_Fe_1–*x*_O_3_ perovskites within the range 5–50° 2θ; Figure S2: Rietveld refinements of different perovskites (a) LaFeO_3_ (b) LaNi_0.1_Fe_0.9_O_3_ (c) LaNi_0.5_Fe_0.5_O_3_ (d) LaNi_0.9_Fe_0.1_O_3_ (e) LaNiO_3_;. Figure S3: PDF refinements of different perovskites (a) LaFeO_3_ (b) LaNi_0.1_Fe_0.9_O_3_ (c) LaNi_0.5_Fe_0.5_O_3_ (d) LaNi_0.9_Fe_0.1_O_3_ (e) LaNiO_3_; Table S2: Refined parameters obtained after the final PDF refinement of LaNi_*x*_Fe_1–*x*_O_3_ perovskites within the range 1.5–15 Å; Figure S4: High-resolution transmission electron microscopy images of (a) & (b) LaNiO_3_, (c) & (d) LaNi_0.5_Fe_0.5_O_3,_ and (e) & (f) LaFeO_3_; Figure S5: Raman modes for different LaNi_*x*_Fe_1–*x*_O_3_ perovskites; Table S3: Vibrational modes corresponding to LaNi_*x*_Fe_1–x_O_3_ perovskites; Figure S6: (a) Linear sweep voltammetry of different LaNi_*x*_Fe_1–*x*_O_3_ perovskites and (b) chronopotentiometry of LaNi_0.5_Fe_0.5_O_3_ at 10 mA/cm^2^ in 1 M KOH; Table S4: Fittings of the impedance spectra of different LaNi_*x*_Fe_1–*x*_O_3_ perovskites; Figure S7: TEM images of LaNi_0.5_Fe_0.5_O_3_ (a) before and (c) after the stability test and SEM images of LaNi_0.5_Fe_0.5_O_3_ (b) before and (d) after the stability test; Figure S8: Raman modes for different LaNi_*x*_Fe_1–*x*_O_3_ perovskites at 1.5 V vs RHE; Figure S9: HAADF-STEM images, where the regions analyzed by EELS for the LaNi_0.5_Fe_0.5_O_3_ are highlighted by a colored square (a) before OER, (b) after OER in pure KOH,.(c) EELS data showing O K-edge, Fe L-edge, and La M-edge; Figure S10: Raman spectra of LaNi_0.5_Fe_0.5_O_3_ before and after OER; Figure S11: Cyclic voltammetry of LaFeO_3_ in 1 M KOH with different Ni impurities; Table S5: Concentration of Ni in different electrolytes used measured using ICP OES; Figure S12: STEM images of the LaFeO_3_ (a) before OER, (b) after OER in pure KOH, (c) after OER in the presence of Ni impurities; Figure S13: HAADF-STEM images, where the regions analyzed by EELS for the LaFeO_3_ system are highlighted by a colored square (a) Before OER, (b) after OER in pure KOH, (c) after OER in pure KOH+ Ni impurity;. Figure S14: Thickness over mean free path maps from the EELS data of LaFeO_3_ (a) before OER, (b) after OER in pure KOH, and (c) in the presence of Ni impurities in the electrolyte; Figure S15: Zero loss peak showing a similar full width half-maximum (and energy resolution) for the EELS data before OER (blue), after OER in pure KOH (green), and in the presence of Ni impurities in the electrolyte (red) (PDF)

## Abbreviations

OER: oxygen evolution reaction
XRD: X-ray diffraction
PDF: pair distribution function
SERS: surface enhanced Raman spectroscopy
STEM: scanning transmission electron microscopy
EELS: electron energy loss spectroscopy
SEM: scanning electron microscopy
TEM: transmission electron microscopy
HAADF: high -angle annular dark field
