# Supplementary material for: Unraveling the Evolution of Dynamic Active Sites of LaNixFe1–xO3 Catalysts During OER
Source: ACS Appl Mater Interfaces. 2024 Apr 22;16(17):21997–2006. doi: 10.1021/acsami.4c02502 (PMC11071036; doi:10.1021/acsami.4c02502)
Supplement: Supplementary file 1 — am4c02502_si_001.pdf [file am4c02502_si_001.pdf]

Supporting Information

Unraveling the evolution of dynamic active sites of $LaNi_xFe_{1-x}O_3$ catalysts during OER

Haritha Cheraparambil,[a] Miquel Vega-Paredes,[b] Christina Scheu,[b] Claudia Weidenthaler[a]*

[a]Max-Planck-Institut für Kohlenforschung, Kaiser-Wilhelm-Platz 1, 45470 Mülheim an der Ruhr, Germany

[b]Max-Planck-Institut für Eisenforschung, Max-Planck-Straße 1, 40237 Düsseldorf, Germany

Corresponding author: weidenthaler@mpi-muelheim.mpg.de.

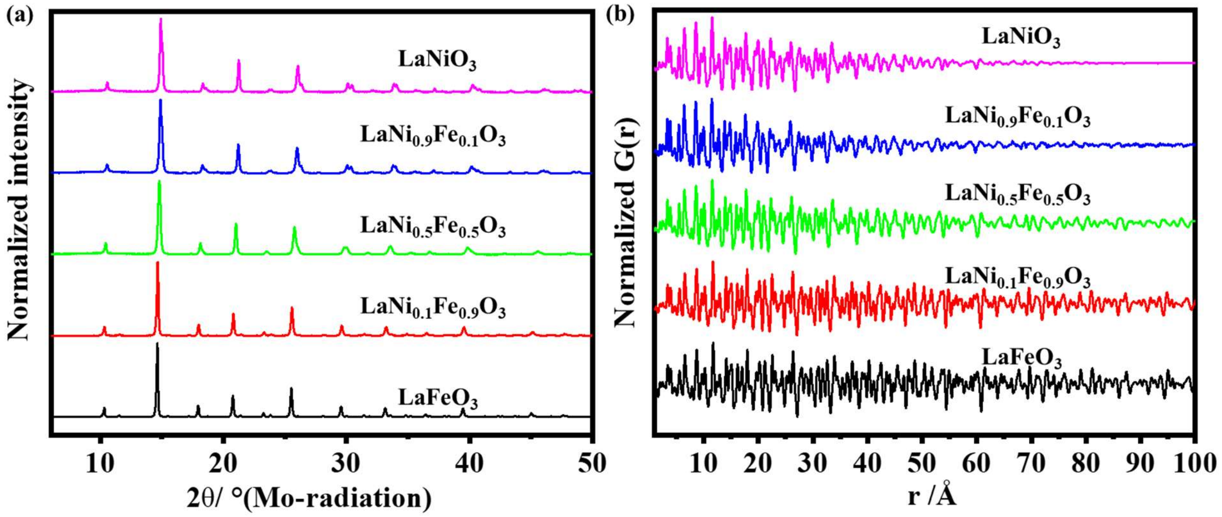

Figure S1 (a) X-ray diffraction patterns, (b) long-range experimental PDFs of different $LaNi_xFe_{1-x}O_3$ perovskites.

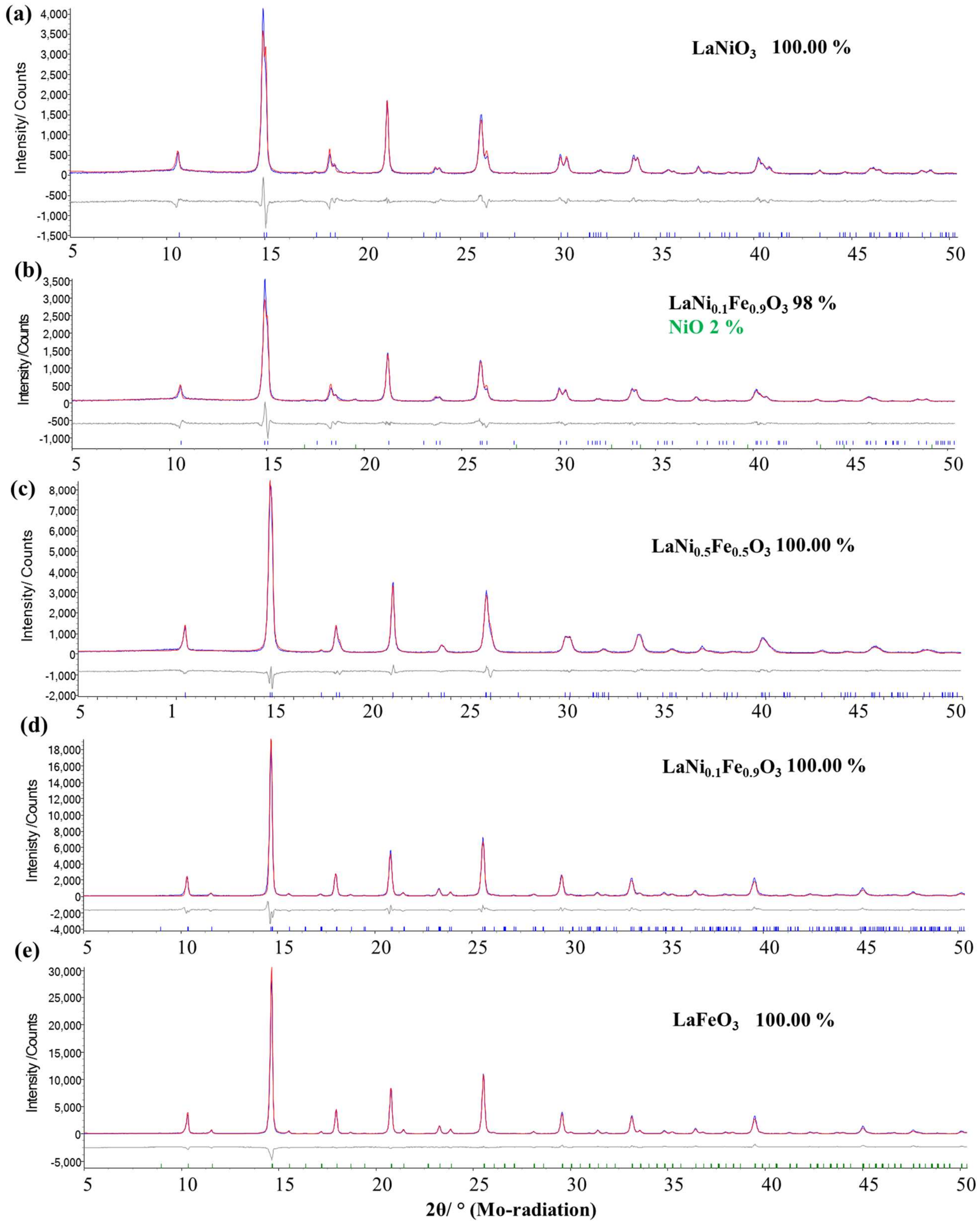

Figure S2 Rietveld refinement plots obtained for different perovskites (a) $LaNiO_3$ (b) $LaNi_{0.9}Fe_{0.1}O_3$ (c) $LaNi_{0.5}Fe_{0.5}O_3$ (d) $LaNi_{0.1}Fe_{0.9}O_3$ (e) $LaFeO_3$.

Table S1 Refined parameters obtained after the final Rietveld refinement of $LaNi_xFe_{1-x}O_3$ perovskites within the range 5-50° 2θ. (Profile function-TCHZ was refined, Debye Waller factors were fixed to 0.5 Å^2, background was refined to 2nd order, and the simple axial model was used)

Perovskites	Initial parameters	Lattice parameters/ Å	Space group	Rwp/ %	Atomic coordinates
$LaNiO_3$	a=b= 5.4534<br>c= 13.1369	a=b= 5.4535(2)<br>c= 13.1602(8)	Rhombohedral, *R-3c H*	16.0	O1 0.452 (2), 0, 1/4
$LaNi_{0.9}Fe_{0.1}O_3$	a=b= 5.4670<br>c= 13.1694	a=b= 5.4647(3)<br>c= 13.1868(9)	Rhombohedral, *R-3c H*	12.1	O1 0.544(2), 0, 1/4
$LaNi_{0.5}Fe_{0.5}O_3$	a=b=5.5199<br>c=13.2842	a=b=5.506(1)<br>c=13.337(3)	Rhombohedral, *R-3c H*	16.5	O1 0.557 (2), 0, 1/4
$LaNi_{0.1}Fe_{0.9}O_3$	a= 5.5485<br>b= 7.8401<br>c= 5.5495	a= 5.5361(3)<br>b= 7.8186(4)<br>c= 5.5610(2)	Orthorhombic, *Pnma*	12.1	La1 0.0227(2), ¼, -0.0024(7)<br>O1 0.488(2), ¼, 0.059(3)<br>O2 0.277(3), 0.0391(19) 0.726(3)
$LaFeO_3$	a= 5.7677<br>b= 7.8591<br>c= 5.554	a= 5.5607(6)<br>b= 7.853(4)<br>c= 5.558(1)	Orthorhombic, *Pbnm*	16.8	La1 0.0283(2), ¼, -0.0106(3)<br>O1 0.480(2), ¼, 0.050(4)<br>O2 0.293(5), 0.031(2), 0.713(5)

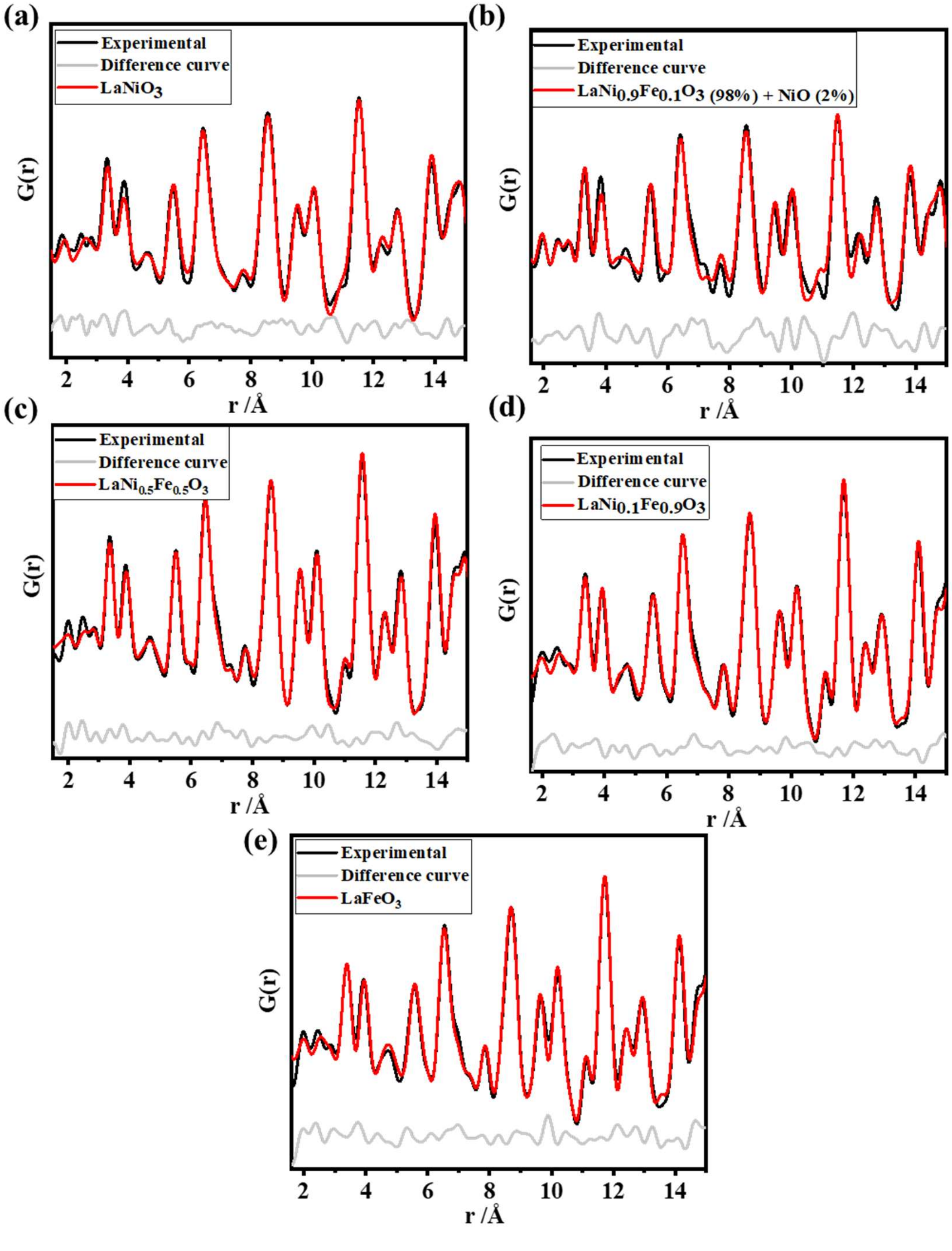

Figure S3 PDF refinement plots obtained for the different perovskites (a) $LaNiO_3$ (b) $LaNi_{0.9}Fe_{0.1}O_3$ (c) $LaNi_{0.5}Fe_{0.5}O_3$ (d) $LaNi_{0.1}Fe_{0.9}O_3$ (e) $LaFeO_3$.

Table S2 Refined parameters obtained after the final PDF refinement of $LaNi_xFe_{1-x}O_3$ perovskites within the range 1.5-15 Å.

	Initial parameters	$LaNiO_3$	$LaNi_{0.9}Fe_{0.1}O_3$	$LaNi_{0.5}Fe_{0.5}O_3$	$LaNi_{0.1}Fe_{0.9}O_3$	$LaFeO_3$
Lattice parameters/ Å		a=b=5.45 (0.003) c=13.18 (0.021)	a=b= 5.49 (0.002) c=13.19 (0.013)	a=b=5.52 (0.006) c=13.36 (0.032)	a=5.56 (0.016) b=7.83(0.027) c=5.55 (0.018)	a=5.60(0.023) b=5.57(0.022) c=7.82(0.027)
Atomic displacement factor/ $Å^2$	La-0.005 Fe-0.005 Ni-0.005 O-0.005	0.005(0.0004) 0.006(0.0008) 0.037(0.009)	0.012 (0.009) 0.016 (0.008) 0.008 (0.001) 0.014 (0.005)	0.012 (0.001) 0.018 (0.009) 0.008 (0.002) 0.03 (0.008)	0.010 (0.001) 0.074 (0.006) 0.005 (0.002) 0.095 (0.03)	0.012 (0.003) 0.004 (0.001) 0.034 (0.010)
Quadratic corr. factor	0.5	1.10	1.83	0.38	1.06	1.60
Linear corr. factor	0.5	0.8	0.98	0.29	0.9	0.68
R_{wp}/ %		12	23	14	10	7

Table

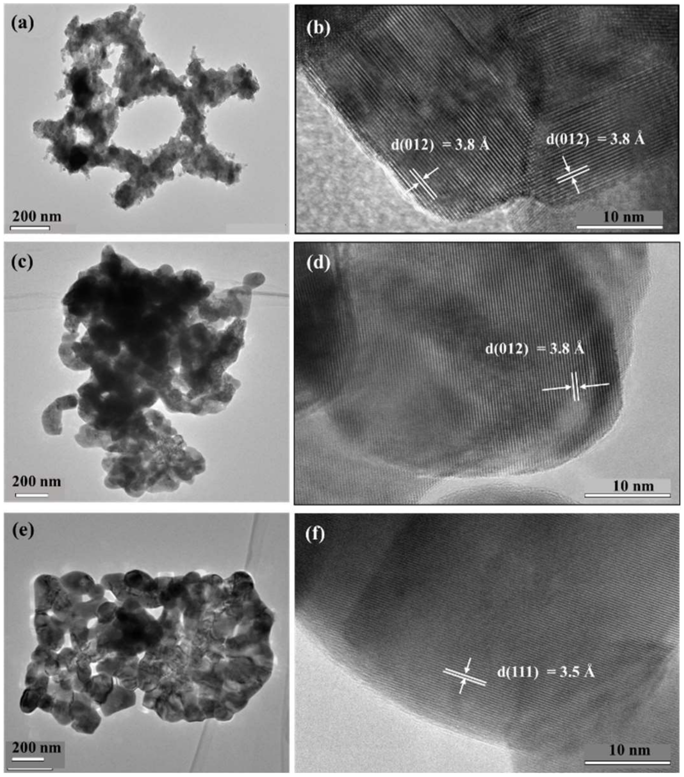

Figure S4 High-resolution transmission electron microscopy images of (a) & (b) $LaNiO_3$, (c) & (d) $LaNi_{0.5}Fe_{0.5}O_3$, and (e) & (f) $LaFeO_3$.

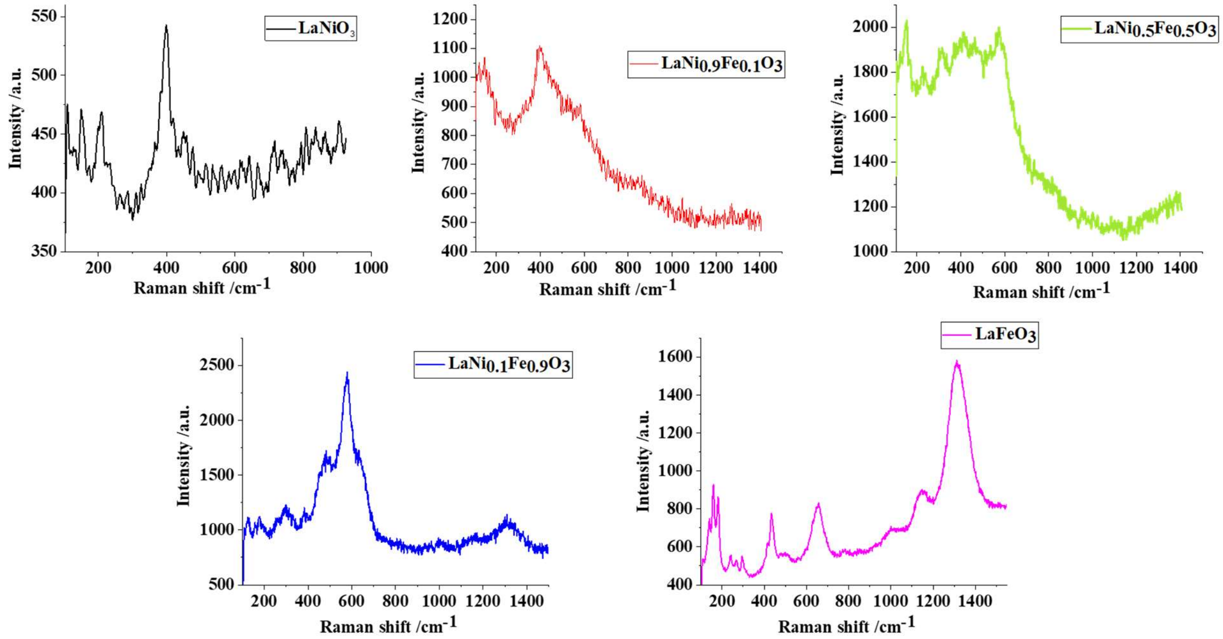

Figure S5 Ex situ Raman spectroscopy of different $LaNi_xFe_{1-x}O_3$ perovskites (532 nm Raman laser).

Table S3 Vibrational modes corresponding to $LaNi_xFe_{1-x}O_3$ perovskites.

Raman shift/ cm^{-1}	Vibrational modes
>200	Vibrational modes of La
250-300	Octahedra tilts
400-625	Stretching and bending modes of the octahedra
>700	Multiphoton processes

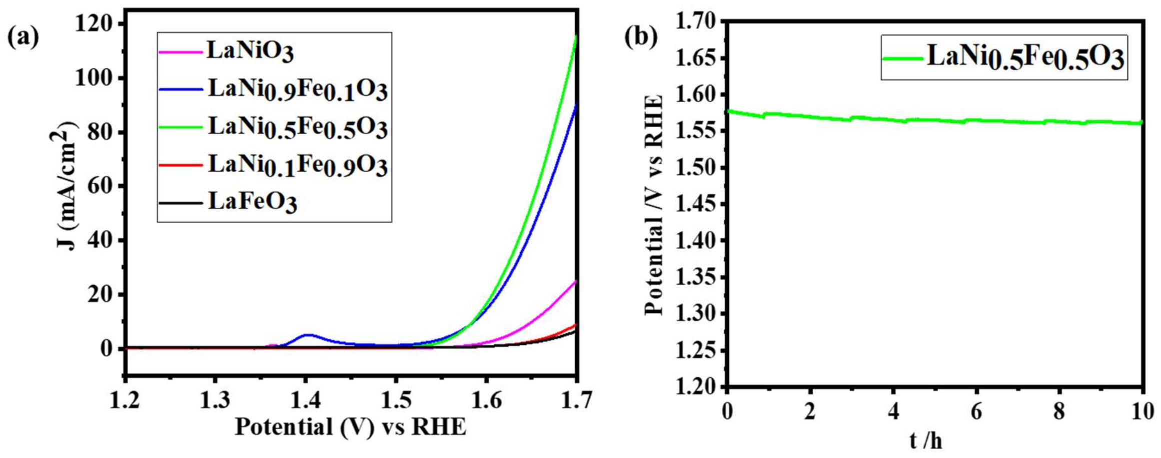

Figure S6 (a) Linear sweep voltammetry of different $LaNi_xFe_{1-x}O_3$ perovskites (b) chronopotentiometry of $LaNi_{0.5}Fe_{0.5}O_3$ at 10 mA/cm^2 in 1M KOH.

Table S4 Fittings of the impedance spectra of different $LaNi_xFe_{1-x}O_3$ perovskites.

**Samples**	**$R_{electrolyte}$ (Ω)**	**R_{ct} (Ω)**
$LaNiO_3$	1.3	6.5
$LaNi_{0.9}Fe_{0.1}O_3$	1.3	2.4
$LaNi_{0.5}Fe_{0.5}O_3$	1.5	1.2
$LaNi_{0.1}Fe_{0.9}O_3$	1.5	15.9
$LaFeO_3$	1.6	30.1

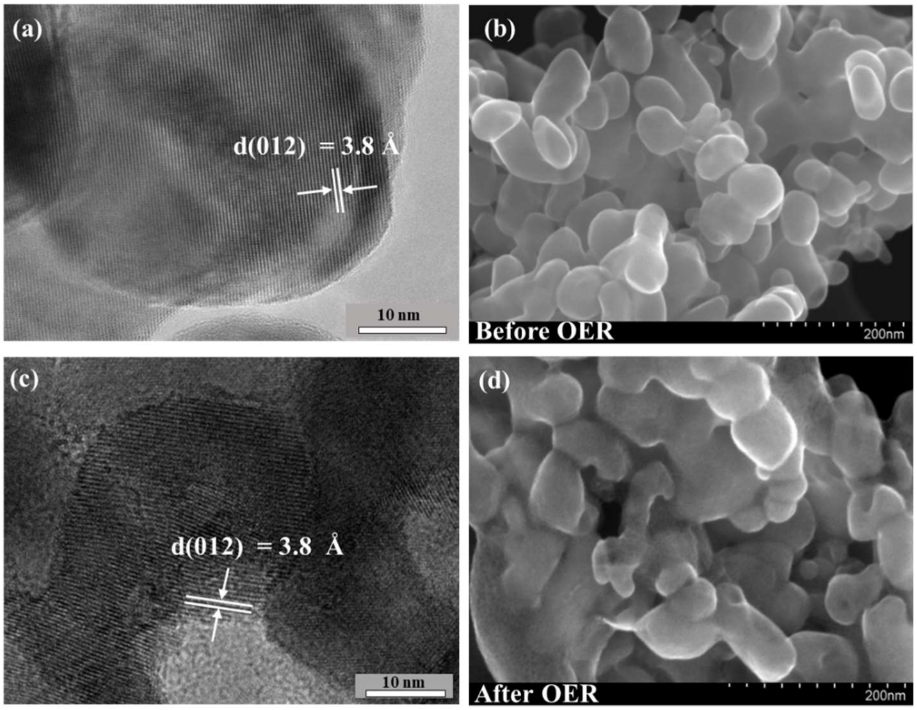

Figure S7 TEM images of $LaNi_{0.5}Fe_{0.5}O_3$ (a) before and (c) after the stability test. SEM images of $LaNi_{0.5}Fe_{0.5}O_3$ (b) before and (d) after the stability test.

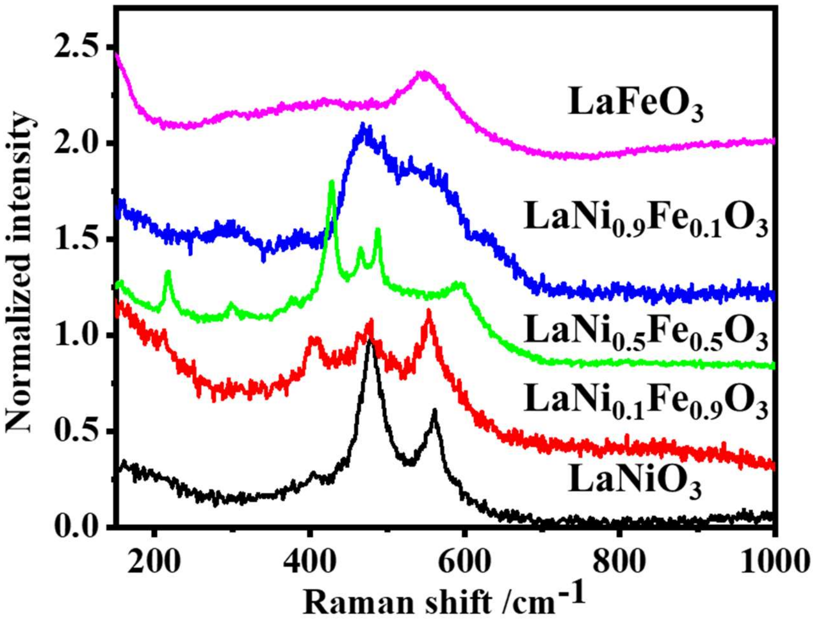

Figure S8 Raman modes for different $LaNi_xFe_{1-x}O_3$ perovskites at 1.5 V vs RHE (using 785 nm laser).

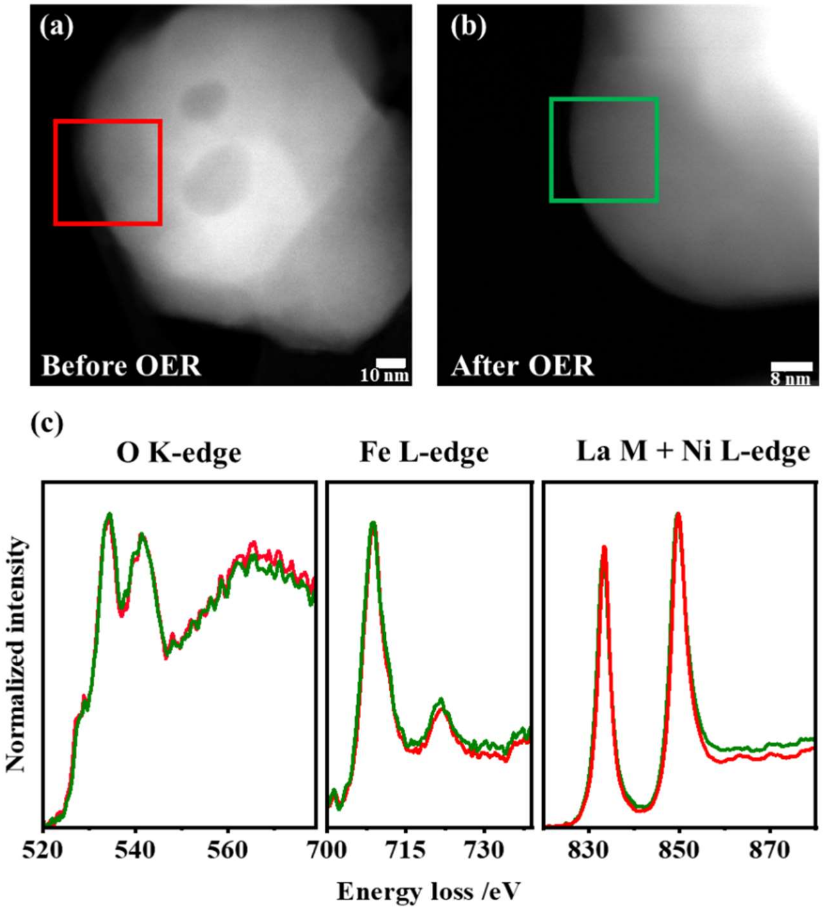

Figure S9 HAADF-STEM images, where the regions analyzed by EELS for the $LaNi_{0.5}Fe_{0.5}O_3$ are highlighted by a colored square. (a) Before OER, (b) after OER in pure KOH (c) EELS data showing O K-edge, Fe L-edge, and La M-edge.

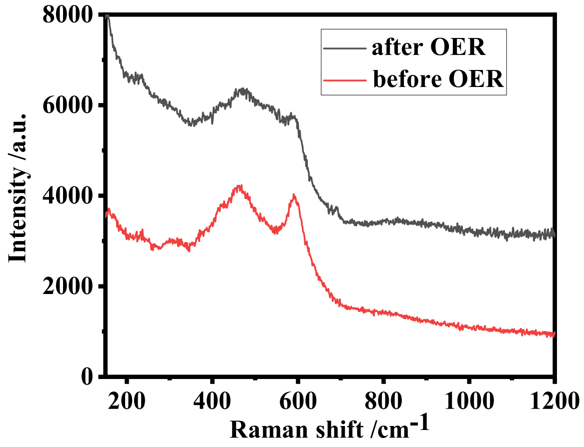

Figure S10 In situ Raman spectra obtained for $LaNi_{0.5}Fe_{0.5}O_3$ before and after OER (using 785 nm laser).

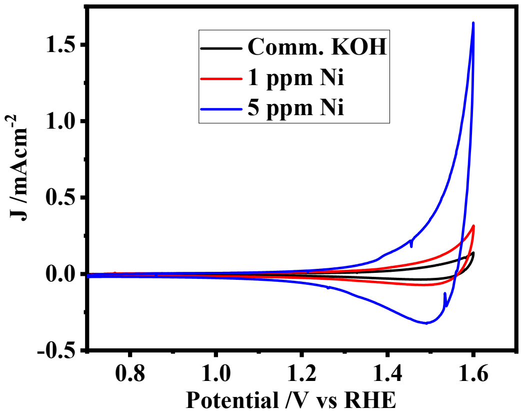

Figure S11 Cyclic voltammetry of $LaFeO_3$ in 1M KOH with different Ni impurities.

Table S5 Concentration of Ni in different electrolytes used measured using ICP OES.

Electrolyte	Ni (ppm)	Fe (ppm)
Comm. KOH	0.01	0.36
Semiconductor grade KOH/ Pure KOH	0.01	0.01
Pure KOH+ 1 ppm Ni	0.99	0.01
Pure KOH+ 5 ppm Ni	4.56	0.01

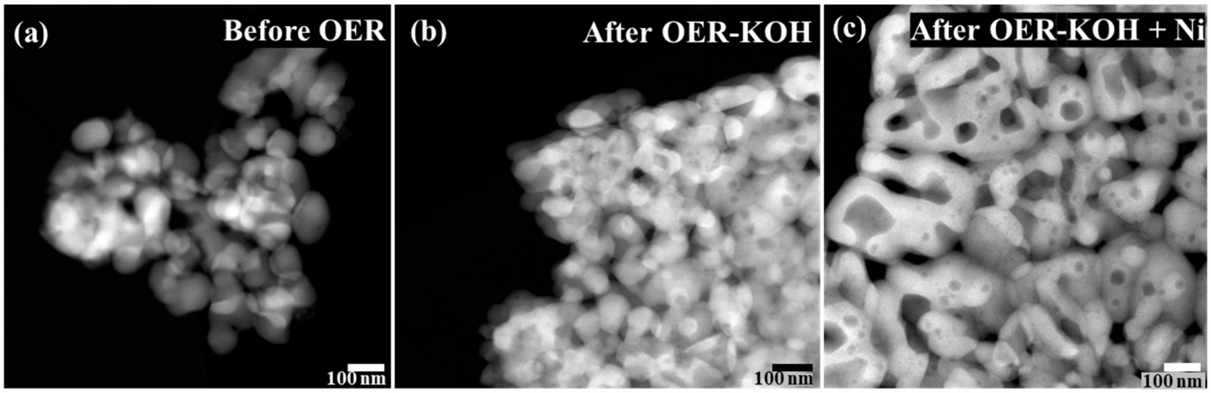

Figure S12 STEM images of the $LaFeO_3$ (a) before OER (b) after OER in pure KOH (c) after OER in the presence of Ni impurities.

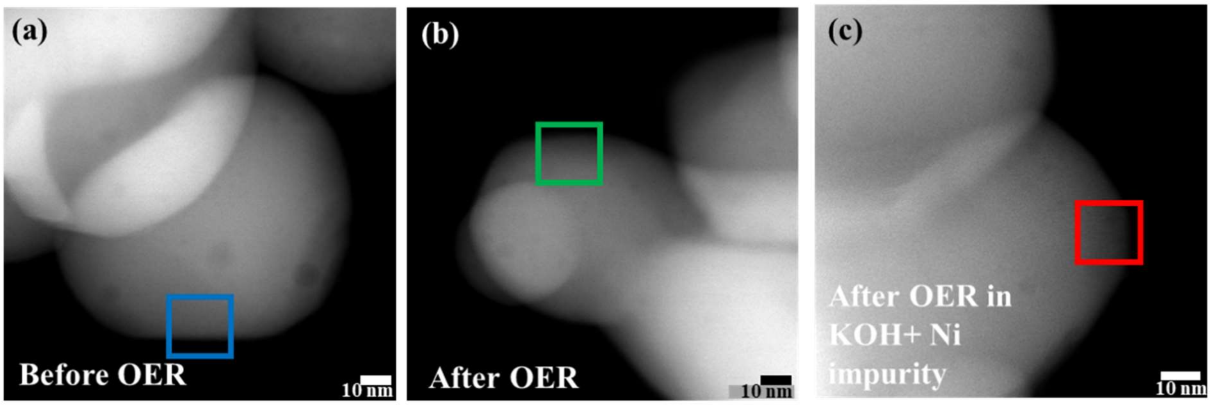

Figure S13 HAADF-STEM images, where the regions analyzed by EELS for the $LaFeO_3$ system are highlighted by a colored square. (a) Before OER, (b) after OER in pure KOH (c) after OER in pure KOH+ Ni impurity.

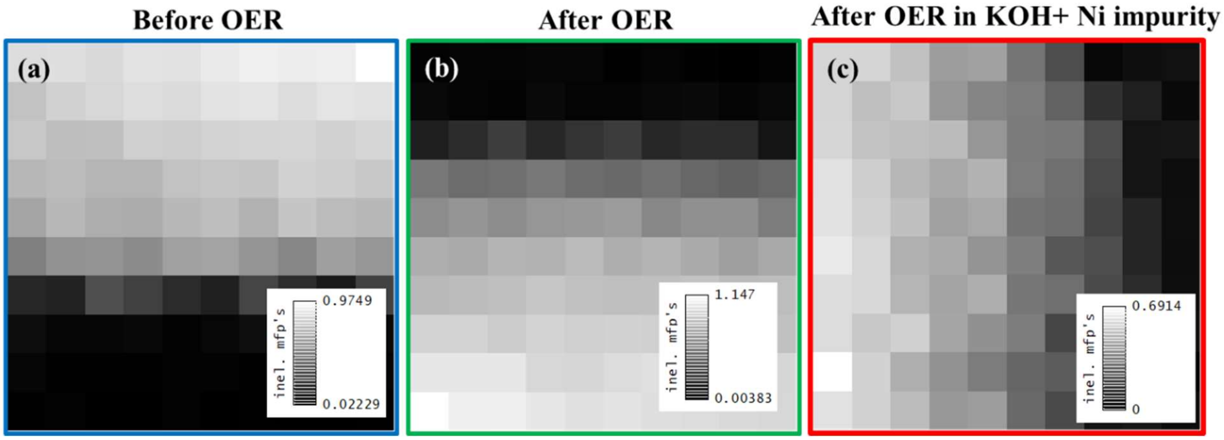

Figure S14 Thickness over mean free path maps from the EELS data of $LaFeO_3$ (a) before OER, (b) after OER in pure KOH (c), and in the presence of Ni impurities in the electrolyte.

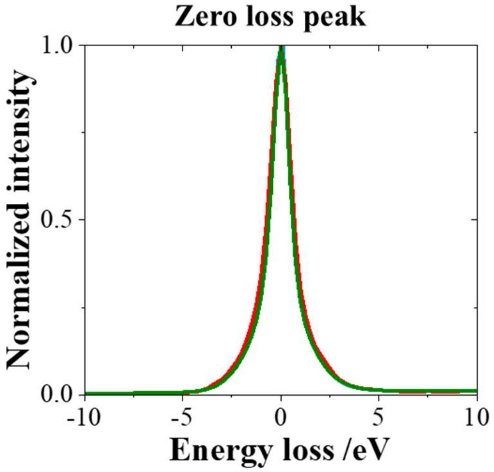

Figure S15 Zero loss peak showing a similar full-width half maximum (and energy resolution) for the EELS data before OER (blue), after OER in pure KOH (green), and in the presence of Ni impurities in the electrolyte (red).
